# Synthesis, crystal structure and Hirshfeld surface analysis of 5-oxo-*N*-phenyl-3-(thio­phen-2-yl)-2,3,4,5-tetra­hydro-[1,1′-biphen­yl]-4-carboxamide

**DOI:** 10.1107/S205698902500057X

**Published:** 2025-01-28

**Authors:** Khatira N. Aliyeva, Tuncer Hökelek, Victor N. Khrustalev, Rovnag M. Rzayev, Ajaya Bhattarai, Abel M. Maharramov, Namig G. Shikhaliyev

**Affiliations:** a Department of Chemistry, Baku State University, Z. Xalilov Str. 23, AZ 1148 Baku, Azerbaijan; b Hacettepe University, Department of Physics, 06800 Beytepe-Ankara, Türkiye; c Friendship University of Russia (RUDN University), Miklukho-Maklay St. 6, Moscow 117198, Russian Federation; d N. D. Zelinsky Institute of Organic Chemistry RAS, Leninsky Prosp. 47, Moscow 119991, Russian Federation; e ‘Composite materials’ Scientific Research Center, Azerbaijan State Economic University (UNEC), Murtuza Mukhtarov Str. 194, AZ 1065, Baku, Azerbaijan; f Department of Chemistry, M. M. A. M. C. (Tribhuvan University), Biratnagar, Nepal; g Department of Chemical Engineering, Baku Engineering University, Hasan Aliyev Str. 120, AZ 0101 Baku, Azerbaijan; Vienna University of Technology, Austria

**Keywords:** crystal structure, carboxamide, hydrogen bond, thio­phene disorder, biphenyl-4-carboxamide

## Abstract

The asymmetric unit of the title com­pound, C_23_H_19_NO_2_S, contains two mol­ecules that differ in the conformation of the two carb­ox­amide moieties. In the crystal, inter­molecular N—H⋯O hydrogen bonds link the mol­ecules into chains propagating parallel to the *c*-axis direction. Between the mol­ecules, weak C—H⋯π(ring) inter­actions are present, whereas π–π inter­actions are not observed. The Hirshfeld surface analysis of the crystal structure indicates that the most important contributions to the crystal packing are from H⋯H (47.6%), H⋯C/C⋯H (33.4%) and H⋯O/O⋯H (11.6%) inter­actions. Orientational disorder is observed for both thio­phene rings.

## Abstract 2

The asymmetric unit of the title biphenyl-4-carboxamide contains two mol­ecules. In the crystal, inter­molecular N—H⋯O hydrogen bonds link the mol­ecules into chains propagating parallel to the *c*-axis direction.

## 1. Chemical context

The syntheses and structural characterization of heterocyclic com­pounds continue to be of inter­est in organic and medicinal chemistry due to the various applications of these compounds in pharmaceuticals, materials science and catalysis (Askerov *et al.*, 2020 ▸; Karimli *et al.*, 2023 ▸; Khalilov, 2021 ▸; Khalilov *et al.*, 2024 ▸). Among these, biphenyl derivatives containing thio­phene and amide functional groups are particularly notable for their biological activities, including anti-inflammatory, anti­cancer and anti­microbial properties (Tas *et al.*, 2023 ▸; Rzayev & Khalilov, 2024 ▸). Furthermore, the structural features of these com­pounds suggest their potential relevance in coordination chemistry (Mahmoudi *et al.*, 2021 ▸; Gurbanov *et al.*, 2021 ▸). In particular, when other functional groups are present, like the amide group, the thio­phene moiety and the biphenyl skeleton, multiple coordination sites are available, enabling the formation of stable metal com­plexes (Khalilov *et al.*, 2018*a* ▸,*b* ▸; Naghiyev *et al.*, 2021*a* ▸,*b* ▸; Akkurt *et al.*, 2018 ▸).
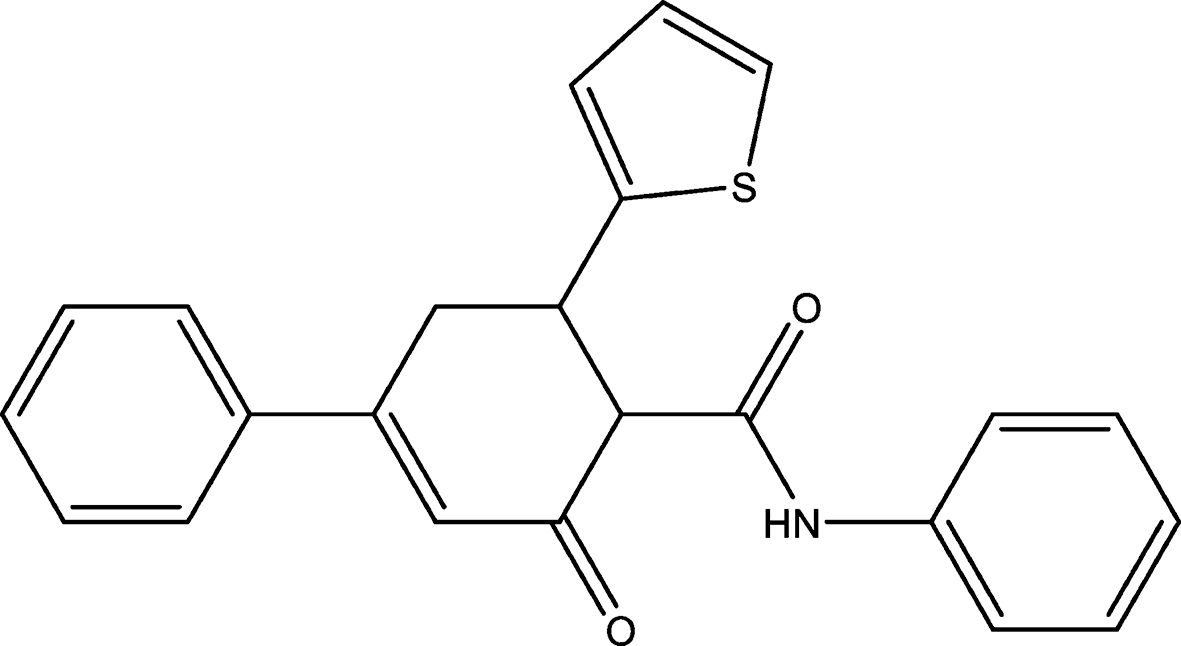


Natural products with a tetra­hydro-[1,1′-biphen­yl] core are rare, but many contain biphenyl-like or partially hydrogenated systems. Examples include flavonoids, stilbenoids and lignans, which often function as biosynthetic inter­mediates or exhibit significant biological activities (Nenajdenko *et al.*, 2023 ▸; Niesen *et al.*, 2013 ▸). Thio­phene-containing fragments appear in natural products such as biotin and thio­cillins, the latter of which exhibit anti­biotic activity. The amide functionality is a common feature in bioactive mol­ecules such as capsaicin, which has pain-relieving properties, and β-lactam anti­biotics, which are critical in medicinal treatments. These structural motifs contribute to the rigidity, conjugation and hydrogen-bonding potential, influencing its inter­actions in biological and chemical environments (Nagiyev *et al.*, 2022 ▸; Mamedov *et al.*, 2020 ▸).

In the context given above, we report here the synthesis, mol­ecular and crystal structures, as well as Hirshfeld surface analysis, of 5-oxo-*N*-phenyl-3-(thio­phen-2-yl)-2,3,4,5-tetra­hydro-[1,1′-biphen­yl]-4-carboxamide. The results provide com­prehensive insights into its mol­ecular shape, hydrogen-bonding inter­actions and crystal packing features, contributing valuable information to the growing database of functionalized carbo- and heterocyclic com­pounds.

## 2. Structural commentary

**Figure 1:**
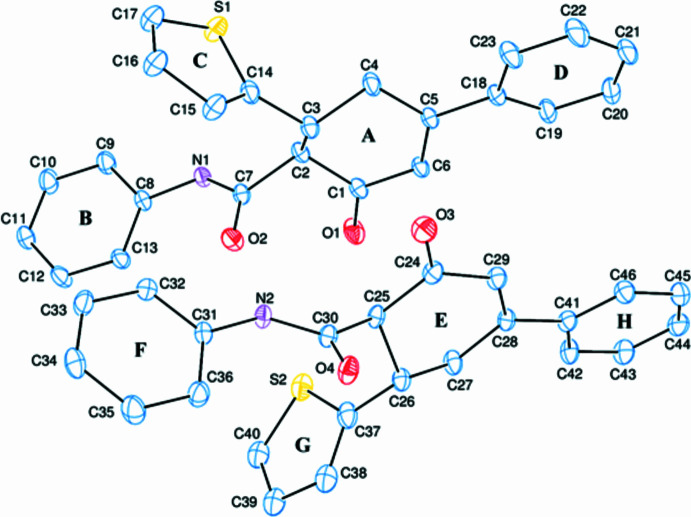
The asymmetric unit of the title com­pound, with displacement ellipsoids drawn at the 50% probability level and with the labelling scheme for the rings. Only the major parts of the disordered thio­phene rings are shown for clarity.

**Figure 2:**
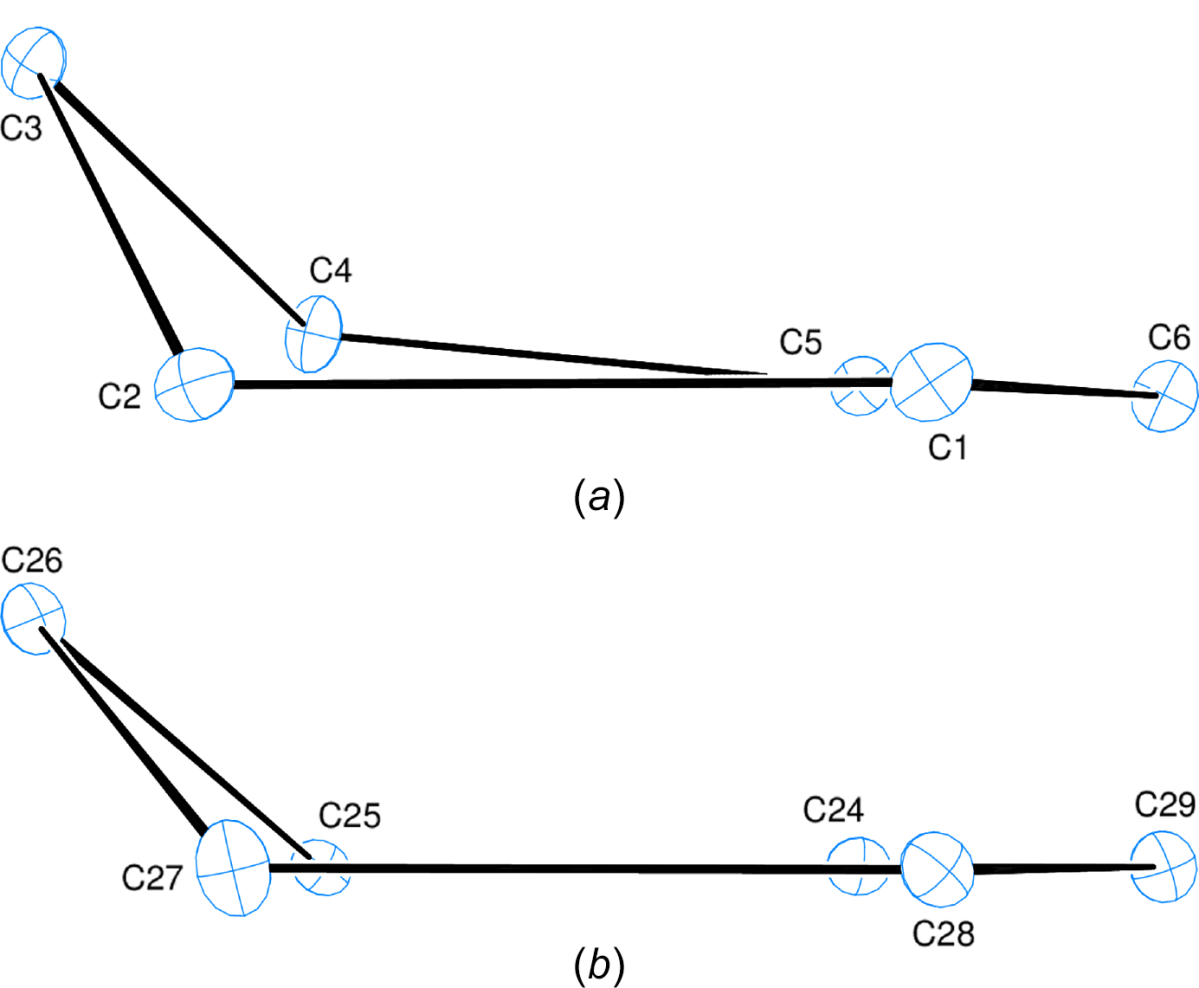
Conformations of the central (*a*) *A* (C1–C6) ring and (*b*) *E* (C24–C29) ring.

**Figure 3:**
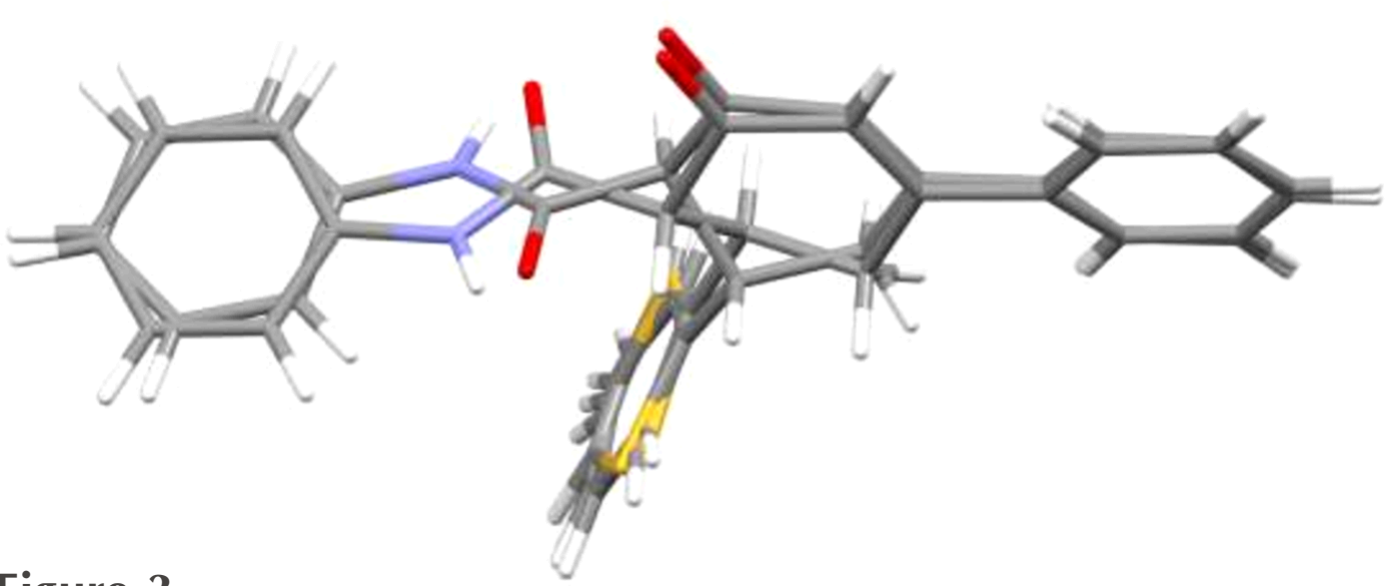
An overlay plot of the two mol­ecules present in the asymmetric unit.

The asymmetric unit of the title com­pound com­prises two mol­ecules (Fig. 1 ▸). The *A* (C1—C6) and *E* (C24—C29) rings are in envelope conformations (Fig. 2 ▸), with puckering parameters (Cremer & Pople, 1975 ▸) of *Q*_T_ = 0.496 (3) Å, θ = 126.0 (3)° and φ = 289.2 (4)° for ring *A*, and *Q*_T_ = 0.443 (3) Å, θ = 126.2 (4)° and φ = 299.2 (4)° for ring *E*, where atoms C3 and C26, respectively, are at the flap positions and are 0.6894 (16) and 0.6191 (16) Å away from the least-squares planes of the other five atoms. The coplanar *B* (C8—C13), *C* (S1/C14—C17) and *D* (C18—C23) rings, and coplanar *F* (C31—C36), *G* (S2/C37—C40) and *H* (C41—C46) rings are oriented at dihedral angles of *B*/*C* = 58.93 (7)°, *B*/*D* = 87.08 (8)° and *C*/*D* = 41.61 (8)°, and *F*/*G* = 62.65 (5)°, *F*/*H* = 89.30 (7)° and *G*/*H* = 80.57 (6)°. Thus, the *B*/*D* and *F*/*H* rings are almost perpendicularly oriented. Both thio­phene rings (*C* and *G*) are disordered over two sets of sites. For a more com­pherensible and visual com­parison of the two mol­ecules present in the asymmetric unit, an overlay plot is given in Fig. 3 ▸. The differences between the two mol­ecules are clearly seen in the conformations about the carb­ox­amide moieties, with torsion angles of 71.4 (3)° for C1—C2—C7—O2 and −60.9 (3)° for C24—C25—C30—O4, so that the N—H and C=O groups in the two mol­ecules are oppositely oriented. The *C* and *D*, and *G* and *H* rings overlap exactly, whereas the *A* and *B*, and *E* and *F* rings do not. There are no unusual bond distances or inter­bond angles in the mol­ecules.

## 3. Supra­molecular features

**Figure 4:**
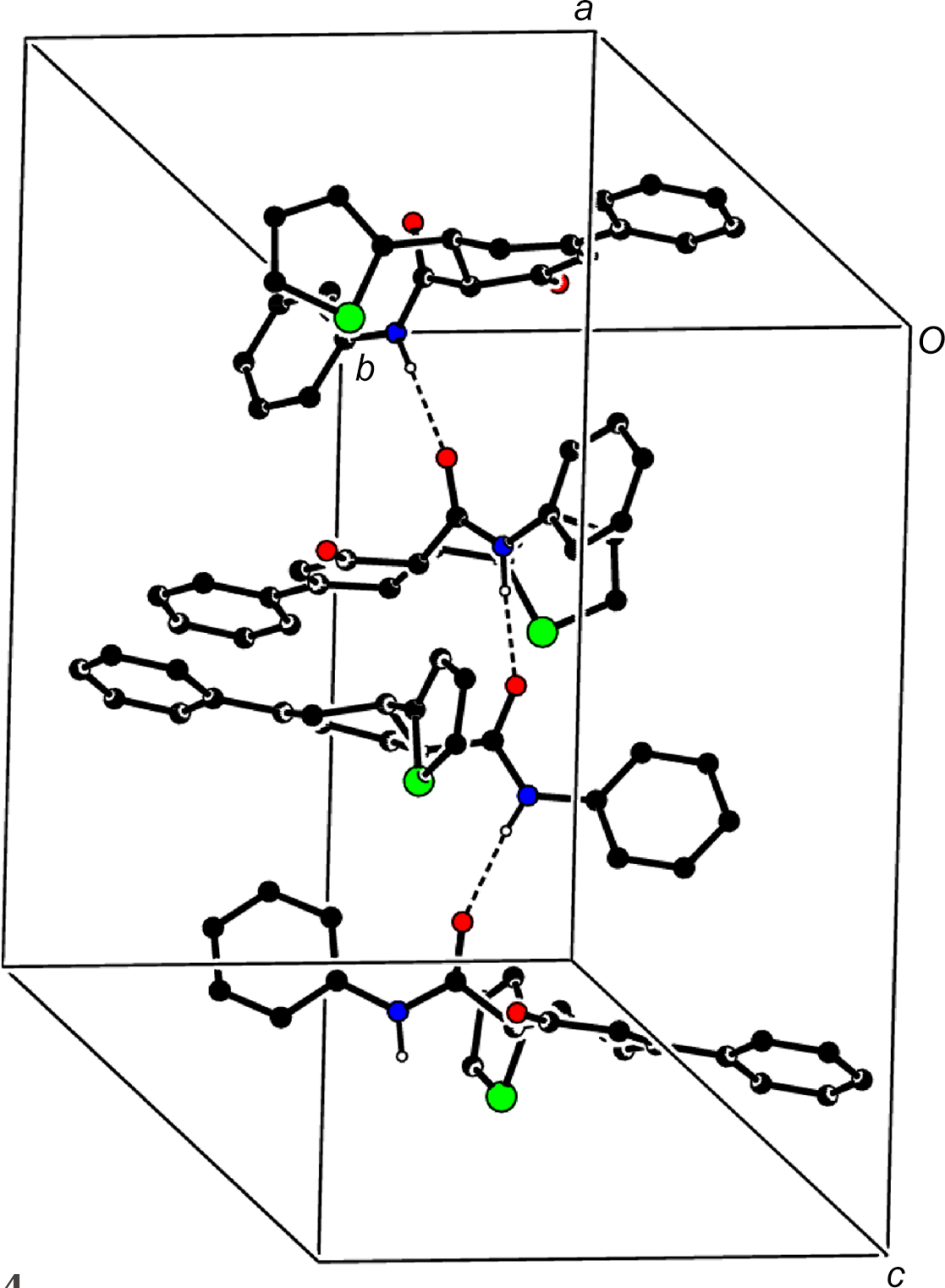
A partial packing diagram showing inter­molecular N—H⋯O hydrogen bonds as dashed lines. Only the major parts of the disordered thio­phene rings are shown for clarity.

In the crystal, inter­molecular N—H⋯O hydrogen bonds between neighbouring carb­ox­amide moieties (Table 1 ▸) link the mol­ecules into supra­molecular chains propagating parallel to the *c*-axis direction (Fig. 4 ▸). Weak C—H⋯π(ring) inter­actions are observed (Table 1 ▸), whereas notable π–π inter­actions are not present.

## 4. Hirshfeld surface analysis

**Figure 5:**
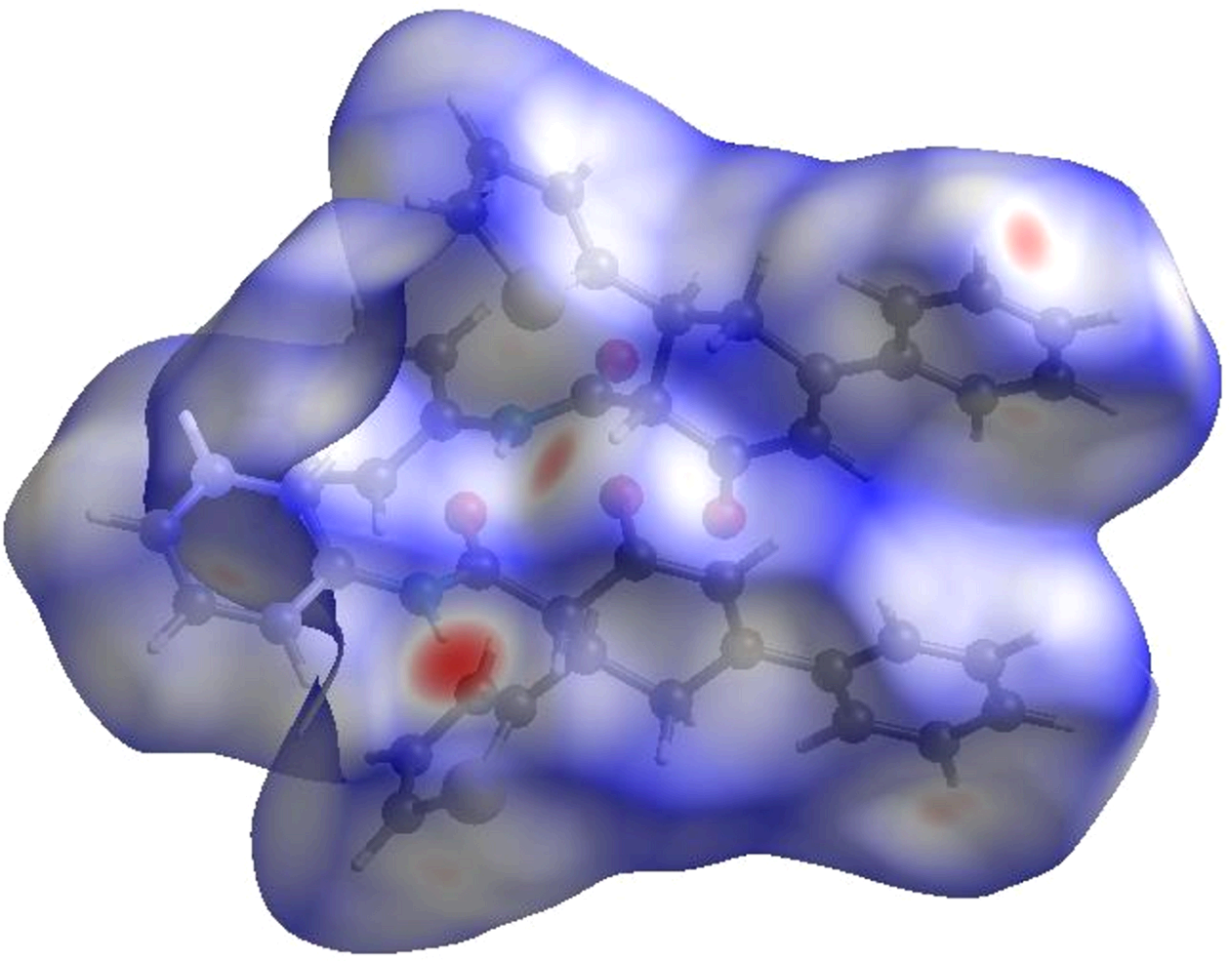
View of the three-dimensional Hirshfeld surface of the title com­pound plotted over *d*_norm_.

**Figure 6:**
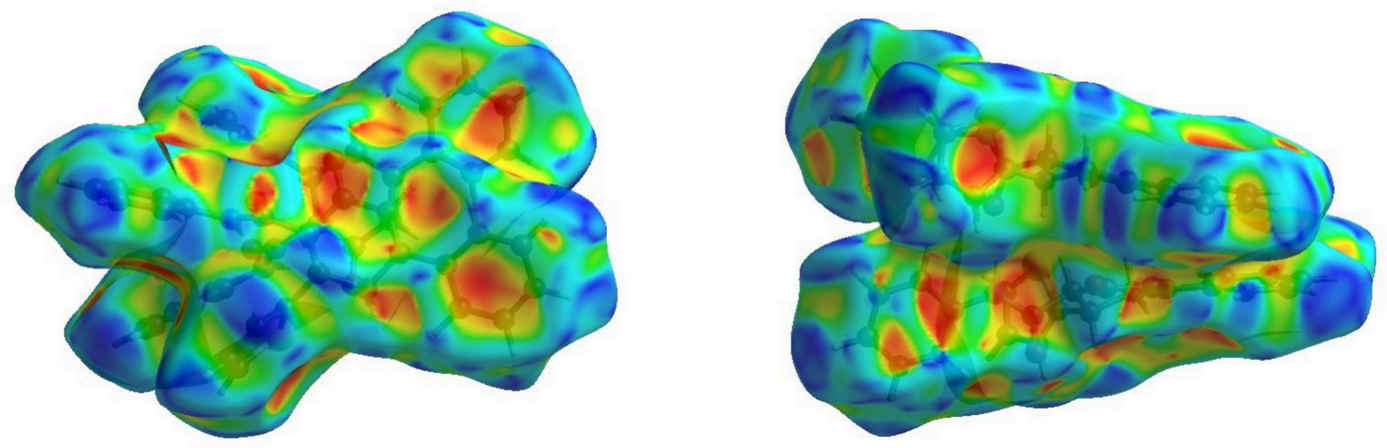
Hirshfeld surface of the title com­pound plotted over shape index for two orientations.

In order to visualize the inter­molecular inter­actions in the crystal of the title com­pound, a Hirshfeld surface (HS) analysis (Hirshfeld, 1977 ▸; Spackman & Jayatilaka, 2009 ▸) was carried out using *CrystalExplorer* (Spackman *et al.*, 2021 ▸). It is noted that only the major com­ponents of the disordered part of the thio­phene rings were taken into account for the analysis. In the HS plotted over *d*_norm_ (Fig. 5 ▸), the white surface indicates contacts with distances equal to the sum of the van der Waals radii, and the red and blue colours indicate distances shorter or longer than the van der Waals radii, respectively (Venkatesan *et al.*, 2016 ▸). The present bright-red spots indicate their roles as the respective donors and/or acceptors in hydrogen bonding, as discussed. In addition, shape index was used to identify possible π–π stacking and C—H⋯π inter­actions, where π–π stacking is indicated by the presence of adjacent red and blue triangles, and C—H⋯π inter­actions as ‘red *p*-holes’ which are related to the electron ring inter­actions between the C—H groups with the centroid of the aromatic rings of neighbouring mol­ecules. Fig. 6 ▸ clearly suggests that there are C—H⋯π inter­actions in the title com­pound but no π–π inter­actions.

**Figure 7:**
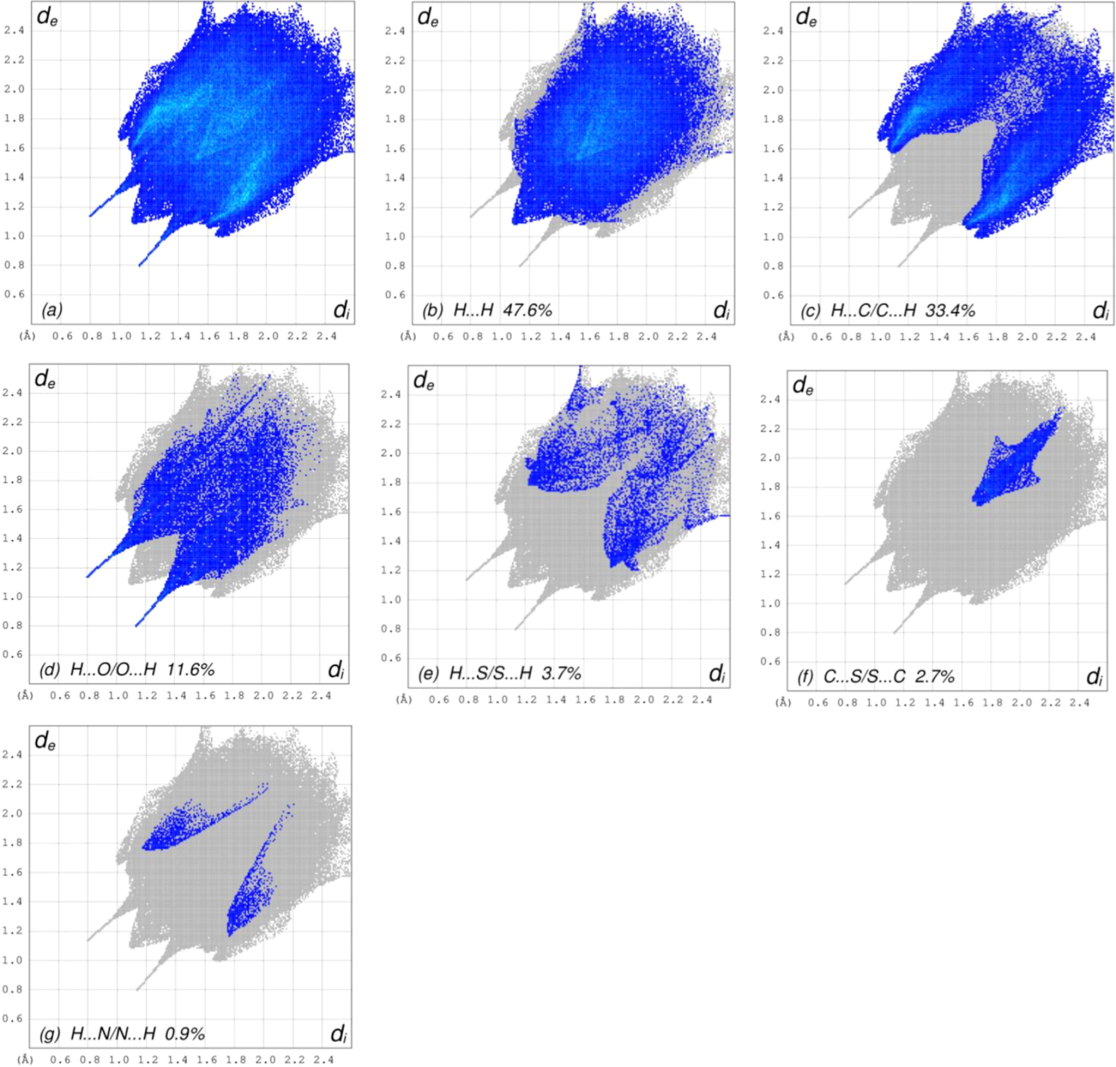
The full two-dimensional fingerprint plots for the title com­pound, showing (*a*) all inter­actions, and delineated into (*b*) H⋯H, (*c*) H⋯C/C⋯H, (*d*) H⋯O/O⋯H, (*e*) H⋯S/S⋯H, (*f*) C⋯S/S⋯C and (*g*) H⋯N/N⋯H inter­actions. The *d*_i_ and *d*_e_ values are the closest inter­nal and external distances (in Å) from given points on the Hirshfeld surface contacts.

The overall two-dimensional fingerprint plot (McKinnon *et al.*, 2007 ▸) is ahown in Fig. 7( ▸*a*) and those delineated into H⋯H, H⋯C/C⋯H, H⋯O/O⋯H, H⋯S/S⋯H, C⋯S/S⋯C and H⋯N/N⋯H inter­actions are illustrated in Figs. 7 ▸(*b*)–(*g*), respectively, together with their relative contributions to the Hirshfeld surface. The most important inter­action is H⋯H (Table 2 ▸), contributing 47.6% to the overall crystal packing, which is reflected in Fig. 7 ▸(*b*), with the tip at *d*_e_ = *d*_i_ = 1.10 Å. Due to C—H⋯π inter­actions (Table 1 ▸), the characteristic wings of the H⋯C/C⋯H contacts (Table 2 ▸) are reflected in Fig. 7 ▸(*c*), with the tips at *d*_e_ + *d*_i_ = 2.64 Å and *d*_e_ + *d*_i_ = 2.66 Å for the sharper and wider ones, respectively. The symmetrical pairs of spikes of the H⋯O/O⋯H [Table 2 ▸ and Fig. 7 ▸(*d*)] and H⋯S/S⋯H [Table 2 ▸ and Fig. 7 ▸(*e*)] contacts are viewed with the tips at *d*_e_ + *d*_i_ = 1.94 Å and *d*_e_ + *d*_i_ = 3.00 Å, respectively. The C⋯S/S⋯C [Fig. 7 ▸(*f*)] contacts have an arrow-shaped distribution of points, and they are viewed with the tip at *d*_e_ = *d*_i_ = 1.72 Å. Finally, the H⋯N/N⋯H [Fig. 7 ▸(*g*)] contacts contribute only marginally to the HS.

**Figure 8:**
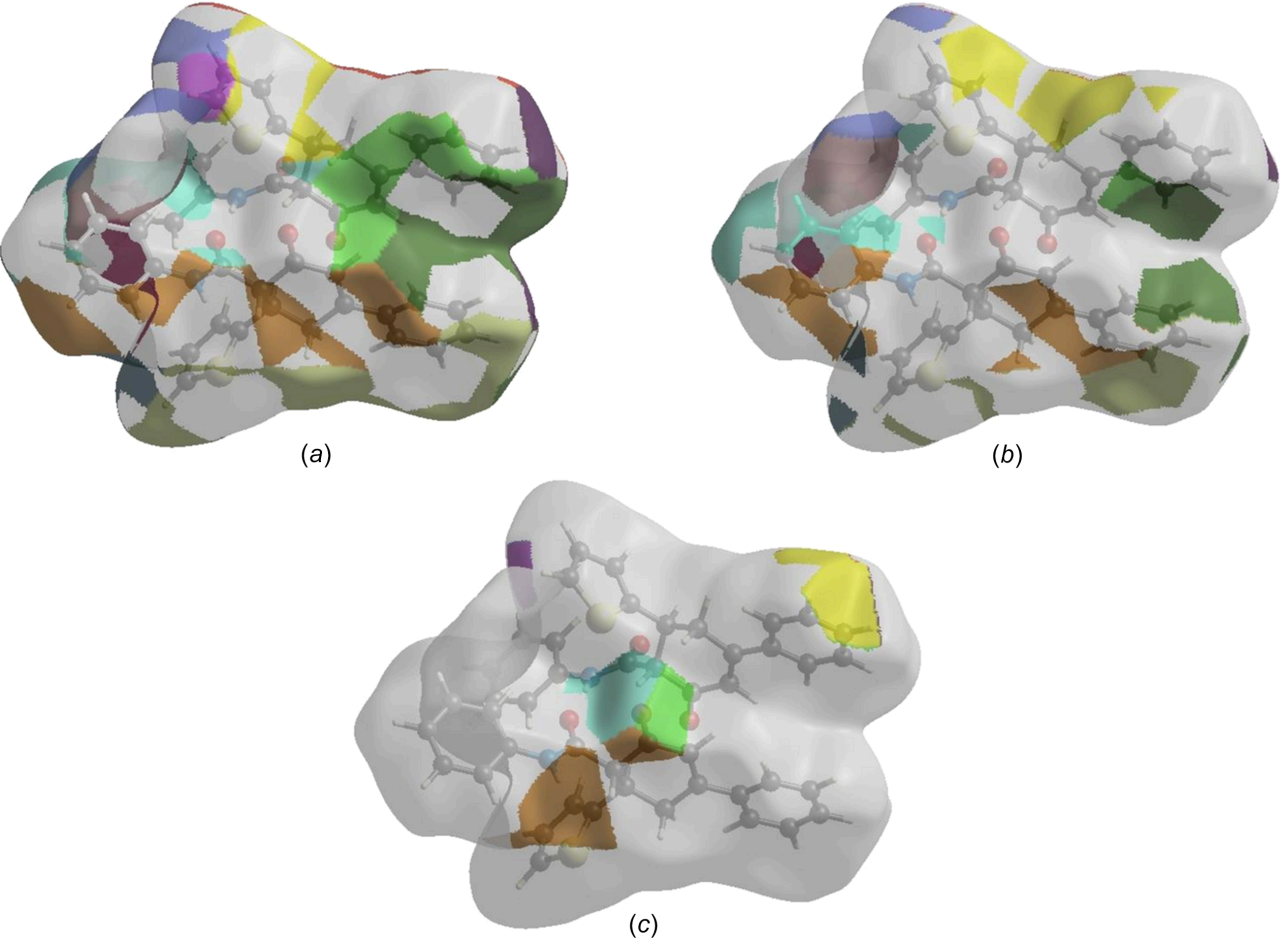
The Hirshfeld surface representations of contact patches plotted onto the surface for (*a*) H⋯H, (*b*) H⋯C/C⋯H and (*c*) H⋯O/O⋯H inter­actions.

The nearest neighbour environment of a mol­ecule can be determined from the colour patches on the HS based on how close to other mol­ecules they are. The Hirshfeld surface representations of contact patches plotted onto the surface are shown for the H⋯H, H⋯C/C⋯H and H⋯O/O⋯H inter­actions in Figs. 8 ▸(*a*)–(*c*), respectively.

In summary, the HS analysis confirms the importance of H-atom contacts in establishing the packing. The large number of H⋯H, H⋯C/C⋯H and H⋯O/O⋯H inter­actions suggest that corresponding van der Waals inter­actions, as well as hydrogen bonding, play the major roles in the crystal packing (Hathwar *et al.*, 2015 ▸).

## 5. Synthesis and crystallization

A solution of acetoacetanilide (5.20 mmol) and 1-phenyl-3-(thio­phen-2-yl)prop-2-en-1-one (5.10 mmol) in methanol (10 ml) was stirred for 1 h. 3 drops of methyl­piperazine were then added to the solution. The resulting mixture was refluxed for 3 h. When the reaction was com­plete, it was kept for 5 d for the formation of crystals, which were separated by filtration and recrystallized from an ethanol–water solution (m.p. 568–569 K; yield: 72%). ^1^H NMR (300 MHz, DMSO-*d*_6_): δ 3.19 (*dd*, 2H, CH_2_), 3.86 (*d*, 1H, CH), 4.16 (*k*, 1H, CH) **[***q*, 6.59 (*s*, 1H, =CH), 6.95–7.74 (*m*, 13H, 10CH_ar_ + 3CH_thien_), 10.23 (*s*, 1H, NH). ^13^C NMR (75 MHz, DMSO-*d*_6_): δ 36.20 (CH_2_), 38.47 (CH), 61.44 (CH), 119.50 (CH=), 119.60 (CH_ar_), 123.91 (CH_thien_), 124.11 (CH_ar_), 124.69 (CH_ar_), 125.19 (CH_thien_), 126.99 (2CH_ar_), 127.29 (CH_thien_), 129.19 (2CH_ar_), 129.34 (2CH_ar_), 131.01 (CH_ar_), 137.82 (C_thien_), 139.14 (C_tert_), 145.82 (C_ar_), 159.18 (C_ar_), 167.72 (–N—C=O), 195.19 (C=O).

## 6. Refinement

Crystal data, data collection and structure refinement details are summarized in Table 3 ▸. The N—H hydrogens were located in a difference Fourier map and refined freely. The C-bound H-atom positions were calculated geometrically at distances of 1.00 (for methine CH), 0.95 (for aromatic CH) and 0.99 Å (for CH_2_), and refined using a riding model with *U*_iso_(H) = 1.2*U*_eq_(C). Both thio­phene rings are found to be disordered over two sets of sites. They were refined with a fixed occupancy ratio of 0.7:0.3 for the major and minor parts.

## Supplementary Material

Crystal structure: contains datablock(s) I, global. DOI: 10.1107/S205698902500057X/wm5747sup1.cif

Structure factors: contains datablock(s) I. DOI: 10.1107/S205698902500057X/wm5747Isup2.hkl

Supporting information file. DOI: 10.1107/S205698902500057X/wm5747Isup4.cml

cover letter. DOI: 10.1107/S205698902500057X/wm5747sup3.txt

CCDC reference: 2418655

Additional supporting information:  crystallographic information; 3D view; checkCIF report

## Figures and Tables

**Table 1:** Hydrogen-bond geometry (Å, °) *Cg*1, *Cg*2, *Cg*7 and *Cg*10 are the centroids of the S1/C14–C17, S2/C37–C49, C18–C23 and C41–C46 rings, respectively.

*D*—H⋯*A*	*D*—H	H⋯*A*	*D*⋯*A*	*D*—H⋯*A*
N1—H1N⋯O4^iii^	0.85 (4)	2.07 (4)	2.921 (3)	173 (4)
N2—H2N⋯O2	0.94 (4)	1.95 (4)	2.871 (3)	167 (3)
C11—H11⋯*Cg*1^v^	0.95	2.71	3.468 (3)	137
C12—H12⋯*Cg*10^vi^	0.95	2.87	3.772 (3)	159
C34—H34⋯*Cg*2^i^	0.95	2.78	3.546 (3)	139
C38—H38⋯*Cg*7^v^	0.95	2.81	3.603 (5)	142

Symmetry codes: (i) 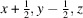; (iii) 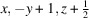; (v) 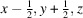; (vi) 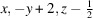.

**Table 2:** Selected interatomic distances (Å)

S1⋯H2	2.95	C23⋯H4*B*	2.57
S2⋯H25	2.96	C27⋯H42	2.54
S2⋯H27*B*	2.93	C27⋯H38*A*	2.97
O2⋯C13	2.920 (3)	C29⋯H46	2.65
O4⋯C36	2.900 (3)	C30⋯H36	2.79
H16⋯O1^i^	2.71	C30⋯H43^i^	2.88
H17*A*⋯O1^i^	2.72	C40⋯H44^iv^	2.84
H22⋯O1^ii^	2.35	C42⋯H27*A*	2.61
O2⋯H3	2.68	C46⋯H29	2.59
O2⋯H13	2.37	H1N⋯H9	2.32
O2⋯H2N	1.95 (4)	H1N⋯H2	2.10
O3⋯H43^i^	2.42	H2N⋯H25	2.03
O4⋯H26	2.67	H4*B*⋯H23	1.96
O4⋯H36	2.38	H6⋯H19	2.06
H1N⋯O4^iii^	2.07 (4)	H27*A*⋯H42	2.11
C4⋯H23	2.61	H27*B*⋯H38*A*	2.37
C6⋯H19	2.62	H29⋯H46	2.05
C7⋯H13	2.84	H40⋯H44^iv^	2.36
C19⋯H6	2.58		

Symmetry codes: (i) 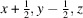; (ii) 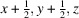; (iii) 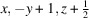; (iv) 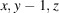.

**Table 3:** Experimental details

Crystal data	
Chemical formula	C_23_H_19_NO_2_S
*M*_r_	373.45
Crystal system, space group	Monoclinic, *Cc*
Temperature (K)	100
*a*, *b*, *c* (Å)	16.1416 (5), 12.8991 (4), 19.0656 (5)
β (°)	107.416 (1)
*V* (Å^3^)	3787.71 (19)
*Z*	8
Radiation type	Mo *K*α
μ (mm^−1^)	0.19
Crystal size (mm)	0.25 × 0.20 × 0.15

Data collection	
Diffractometer	Bruker D8 QUEST PHOTON-III area-detector
Absorption correction	Multi-scan (*SADABS*; Krause *et al.*, 2015 ▸)
*T*_min_, *T*_max_	0.666, 0.746
No. of measured, independent and observed [*I* > 2σ(*I*)] reflections	44070, 13781, 12646
*R*_int_	0.031
(sin θ/λ)_max_ (Å^−1^)	0.758

Refinement	
*R*[*F*^2^ > 2σ(*F*^2^)], *wR*(*F*^2^), *S*	0.050, 0.129, 1.03
No. of reflections	13781
No. of parameters	494
No. of restraints	34
H-atom treatment	H atoms treated by a mixture of independent and constrained refinement
Δρ_max_, Δρ_min_ (e Å^−3^)	0.62, −0.65
Absolute structure	Refined as an inversion twin; Flack *x* determined using 5763 quotients [(*I*^+^)−(*I*^−^)]/[(*I*^+^)+(*I*^−^)] (Parsons & Flack, 2004 ▸)
Absolute structure parameter	0.37 (8)

Computer programs: *APEX3* (Bruker, 2018 ▸), *SAINT* (Bruker, 2018 ▸), *SHELXT* (Sheldrick, 2015*a* ▸), *SHELXL* (Sheldrick, 2015*b* ▸), *SHELXTL* (Sheldrick, 2008 ▸) and *publCIF* (Westrip, 2010 ▸).

## supplementary crystallographic information

### 5-Oxo-N-phenyl-3-(thiophen-2-yl)-2,3,4,5-tetrahydro-[1,1'-biphenyl]-4-carboxamide Crystal data

**Table:**

C_23_H_19_NO_2_S	*F*(000) = 1568
*M**_r_* = 373.45	*D*_x_ = 1.310 Mg m^−^^3^
Monoclinic, *C**c*	Mo *K*α radiation, λ = 0.71073 Å
*a* = 16.1416 (5) Å	Cell parameters from 9482 reflections
*b* = 12.8991 (4) Å	θ = 2.2–32.5°
*c* = 19.0656 (5) Å	µ = 0.19 mm^−^^1^
β = 107.416 (1)°	*T* = 100 K
*V* = 3787.71 (19) Å^3^	Prism, colourless
*Z* = 8	0.25 × 0.20 × 0.15 mm

### 5-Oxo-N-phenyl-3-(thiophen-2-yl)-2,3,4,5-tetrahydro-[1,1'-biphenyl]-4-carboxamide Data collection

**Table:**

Bruker D8 QUEST PHOTON-III area-detector diffractometer	12646 reflections with *I* > 2σ(*I*)
φ and ω scans	*R*_int_ = 0.031
Absorption correction: multi-scan (*SADABS*; Krause *et al.*, 2015)	θ_max_ = 32.6°, θ_min_ = 2.2°
*T*_min_ = 0.666, *T*_max_ = 0.746	*h* = −24→24
44070 measured reflections	*k* = −19→19
13781 independent reflections	*l* = −28→28

### 5-Oxo-N-phenyl-3-(thiophen-2-yl)-2,3,4,5-tetrahydro-[1,1'-biphenyl]-4-carboxamide Refinement

**Table:**

Refinement on *F*^2^	Secondary atom site location: difference Fourier map
Least-squares matrix: full	Hydrogen site location: mixed
*R*[*F*^2^ > 2σ(*F*^2^)] = 0.050	H atoms treated by a mixture of independent and constrained refinement
*wR*(*F*^2^) = 0.129	*w* = 1/[σ^2^(*F*_o_^2^) + (0.0623*P*)^2^ + 3.960*P*] where *P* = (*F*_o_^2^ + 2*F*_c_^2^)/3
*S* = 1.03	(Δ/σ)_max_ = 0.002
13781 reflections	Δρ_max_ = 0.62 e Å^−^^3^
494 parameters	Δρ_min_ = −0.65 e Å^−^^3^
34 restraints	Absolute structure: Refined as an inversion twin; Flack *x* determined using 5763 quotients [(I+)-(I-)]/[(I+)+(I-)] (Parsons & Flack, 2004)
Primary atom site location: difference Fourier map	Absolute structure parameter: 0.37 (8)

### 5-Oxo-N-phenyl-3-(thiophen-2-yl)-2,3,4,5-tetrahydro-[1,1'-biphenyl]-4-carboxamide Special details

**Table:**

Geometry. All esds (except the esd in the dihedral angle between two l.s. planes) are estimated using the full covariance matrix. The cell esds are taken into account individually in the estimation of esds in distances, angles and torsion angles; correlations between esds in cell parameters are only used when they are defined by crystal symmetry. An approximate (isotropic) treatment of cell esds is used for estimating esds involving l.s. planes.
Refinement. Refined as a 2-component inversion twin

### 5-Oxo-N-phenyl-3-(thiophen-2-yl)-2,3,4,5-tetrahydro-[1,1'-biphenyl]-4-carboxamide Fractional atomic coordinates and isotropic or equivalent isotropic displacement parameters (Å^2^)

**Table:**

	*x*	*y*	*z*	*U*_iso_*/*U*_eq_	Occ. (<1)
S1	0.73160 (7)	0.42886 (8)	0.71887 (6)	0.02445 (19)	0.7
S1'	0.73378 (16)	0.38641 (19)	0.57558 (16)	0.0263 (5)	0.3
O1	0.40433 (13)	0.60901 (15)	0.57901 (13)	0.0270 (4)	
O2	0.49029 (13)	0.39952 (15)	0.53972 (10)	0.0221 (3)	
N1	0.48998 (14)	0.37371 (15)	0.65812 (11)	0.0165 (3)	
C1	0.48285 (15)	0.62230 (18)	0.59522 (13)	0.0173 (4)	
C2	0.54702 (15)	0.53526 (17)	0.62719 (13)	0.0163 (4)	
H2	0.564164	0.539595	0.682040	0.020*	
C3	0.62924 (13)	0.54764 (17)	0.60251 (13)	0.0174 (4)	
H3	0.610628	0.545920	0.547561	0.021*	
C4	0.66919 (15)	0.65469 (18)	0.62661 (15)	0.0208 (4)	
H4A	0.693229	0.655867	0.680852	0.025*	
H4B	0.717865	0.665911	0.605888	0.025*	
C5	0.60495 (15)	0.74233 (17)	0.60257 (13)	0.0162 (4)	
C6	0.51878 (15)	0.72339 (17)	0.58679 (13)	0.0168 (4)	
H6	0.479630	0.779177	0.569254	0.020*	
C7	0.50540 (14)	0.42931 (17)	0.60344 (13)	0.0155 (4)	
C8	0.46246 (15)	0.26899 (17)	0.65529 (13)	0.0159 (4)	
C9	0.47950 (19)	0.21882 (19)	0.72294 (14)	0.0232 (5)	
H9	0.508457	0.254912	0.766873	0.028*	
C10	0.4544 (2)	0.1165 (2)	0.72647 (16)	0.0263 (5)	
H10	0.466516	0.082856	0.772774	0.032*	
C11	0.41199 (17)	0.06340 (19)	0.66302 (15)	0.0218 (4)	
H11	0.394683	−0.006549	0.665548	0.026*	
C12	0.39466 (18)	0.1132 (2)	0.59509 (15)	0.0232 (5)	
H12	0.365616	0.076614	0.551390	0.028*	
C13	0.41951 (16)	0.21586 (19)	0.59071 (14)	0.0198 (4)	
H13	0.407405	0.249391	0.544372	0.024*	
C14	0.69198 (9)	0.46161 (14)	0.62909 (10)	0.0199 (4)	
C15	0.7246 (2)	0.3998 (3)	0.5853 (3)	0.02445 (19)	0.7
H15	0.709503	0.406978	0.533395	0.029*	0.7
C16	0.7843 (2)	0.3228 (3)	0.62621 (18)	0.02445 (19)	0.7
H16	0.813366	0.273307	0.605154	0.029*	0.7
C17	0.79315 (18)	0.3309 (2)	0.69932 (19)	0.02445 (19)	0.7
H17	0.829553	0.286942	0.735642	0.029*	0.7
C15'	0.7301 (4)	0.4225 (5)	0.6987 (3)	0.0263 (5)	0.3
H15A	0.715137	0.454323	0.738197	0.032*	0.3
C16'	0.7904 (4)	0.3381 (5)	0.7164 (4)	0.0263 (5)	0.3
H16A	0.819170	0.307482	0.762600	0.032*	0.3
C17'	0.7948 (3)	0.3143 (5)	0.6480 (3)	0.0263 (5)	0.3
H17A	0.830639	0.259395	0.640767	0.032*	0.3
C18	0.63757 (16)	0.84809 (18)	0.59696 (13)	0.0179 (4)	
C19	0.58505 (17)	0.9349 (2)	0.59560 (17)	0.0252 (5)	
H19	0.528169	0.925720	0.599421	0.030*	
C20	0.6148 (2)	1.0343 (2)	0.58875 (19)	0.0313 (6)	
H20	0.578244	1.092451	0.587643	0.038*	
C21	0.6980 (2)	1.0486 (2)	0.58352 (17)	0.0304 (6)	
H21	0.718368	1.116423	0.578379	0.037*	
C22	0.75120 (19)	0.9639 (2)	0.58582 (18)	0.0300 (6)	
H22	0.808301	0.973681	0.582633	0.036*	
C23	0.72128 (18)	0.8642 (2)	0.59279 (17)	0.0251 (5)	
H23	0.758482	0.806550	0.594727	0.030*	
S2	0.28115 (7)	0.47394 (8)	0.41495 (7)	0.02627 (18)	0.7
S2'	0.25927 (18)	0.4669 (2)	0.26876 (14)	0.0280 (5)	0.3
O3	0.59672 (12)	0.67727 (16)	0.42626 (12)	0.0250 (4)	
O4	0.50311 (13)	0.52390 (14)	0.29766 (10)	0.0215 (3)	
N2	0.50923 (14)	0.41810 (15)	0.39523 (11)	0.0163 (3)	
C24	0.51761 (15)	0.68815 (19)	0.41100 (13)	0.0178 (4)	
C25	0.45807 (15)	0.59458 (17)	0.39697 (13)	0.0163 (4)	
H25	0.455743	0.567333	0.445444	0.020*	
C26	0.36501 (13)	0.62122 (17)	0.35019 (13)	0.0181 (4)	
H26	0.366513	0.639001	0.299534	0.022*	
C27	0.33101 (16)	0.71636 (19)	0.38174 (15)	0.0204 (4)	
H27A	0.275071	0.738234	0.346700	0.024*	
H27B	0.319819	0.696322	0.428205	0.024*	
C28	0.39277 (15)	0.80691 (18)	0.39644 (13)	0.0166 (4)	
C29	0.47893 (15)	0.79046 (18)	0.40955 (13)	0.0177 (4)	
H29	0.516314	0.849028	0.418306	0.021*	
C30	0.49336 (15)	0.50904 (17)	0.35838 (13)	0.0165 (4)	
C31	0.53480 (14)	0.32350 (17)	0.36947 (13)	0.0153 (4)	
C32	0.57754 (16)	0.25144 (18)	0.42214 (14)	0.0188 (4)	
H32	0.589395	0.267025	0.472893	0.023*	
C33	0.60284 (17)	0.15704 (19)	0.40074 (15)	0.0221 (4)	
H33	0.632745	0.108574	0.437023	0.026*	
C34	0.58502 (18)	0.13252 (19)	0.32701 (16)	0.0238 (5)	
H34	0.603118	0.067987	0.312537	0.029*	
C35	0.5403 (2)	0.2036 (2)	0.27440 (15)	0.0267 (5)	
H35	0.526614	0.186576	0.223729	0.032*	
C36	0.51516 (19)	0.29968 (19)	0.29508 (14)	0.0226 (5)	
H36	0.485079	0.348121	0.258852	0.027*	
C37	0.30635 (9)	0.53143 (15)	0.34455 (11)	0.0236 (5)	
C38	0.2622 (2)	0.4790 (3)	0.2804 (3)	0.02627 (18)	0.7
H38	0.267720	0.498578	0.233975	0.032*	0.7
C39	0.2081 (2)	0.3941 (3)	0.2878 (2)	0.02627 (18)	0.7
H39	0.174131	0.351909	0.248964	0.032*	0.7
C40	0.21388 (18)	0.3843 (2)	0.36005 (19)	0.02627 (18)	0.7
H40	0.183584	0.332886	0.378302	0.032*	0.7
C38'	0.2831 (4)	0.4893 (5)	0.4023 (4)	0.0280 (5)	0.3
H38A	0.304677	0.517381	0.450437	0.034*	0.3
C39'	0.2257 (4)	0.4022 (5)	0.3880 (4)	0.0280 (5)	0.3
H39A	0.204087	0.365049	0.421800	0.034*	0.3
C40'	0.2093 (3)	0.3847 (4)	0.3147 (3)	0.0280 (5)	0.3
H40A	0.172328	0.330200	0.290280	0.034*	0.3
C41	0.35706 (15)	0.91145 (18)	0.39858 (13)	0.0175 (4)	
C42	0.27141 (16)	0.92410 (19)	0.40067 (15)	0.0211 (4)	
H42	0.236348	0.864781	0.400146	0.025*	
C43	0.23728 (17)	1.0228 (2)	0.40349 (16)	0.0234 (5)	
H43	0.179575	1.030114	0.405746	0.028*	
C44	0.28699 (18)	1.1103 (2)	0.40302 (15)	0.0241 (5)	
H44	0.263395	1.177438	0.404512	0.029*	
C45	0.37189 (18)	1.0989 (2)	0.40036 (15)	0.0230 (4)	
H45	0.406196	1.158652	0.399970	0.028*	
C46	0.40657 (16)	1.00110 (19)	0.39827 (14)	0.0202 (4)	
H46	0.464568	0.994496	0.396597	0.024*	
H1N	0.498 (2)	0.406 (3)	0.699 (2)	0.024*	
H2N	0.496 (2)	0.419 (3)	0.440 (2)	0.024*	

### 5-Oxo-N-phenyl-3-(thiophen-2-yl)-2,3,4,5-tetrahydro-[1,1'-biphenyl]-4-carboxamide Atomic displacement parameters (Å^2^)

**Table:**

	*U*^11^	*U*^22^	*U*^33^	*U*^12^	*U*^13^	*U*^23^
S1	0.0270 (4)	0.0249 (4)	0.0211 (4)	0.0066 (3)	0.0066 (3)	0.0059 (3)
S1'	0.0214 (9)	0.0245 (10)	0.0325 (12)	−0.0058 (8)	0.0073 (7)	−0.0081 (8)
O1	0.0174 (8)	0.0195 (8)	0.0452 (12)	−0.0051 (7)	0.0108 (8)	0.0005 (8)
O2	0.0310 (9)	0.0207 (8)	0.0159 (7)	−0.0068 (7)	0.0091 (7)	−0.0013 (6)
N1	0.0216 (8)	0.0131 (8)	0.0157 (8)	−0.0019 (7)	0.0070 (7)	−0.0006 (6)
C1	0.0171 (9)	0.0155 (9)	0.0210 (10)	−0.0037 (7)	0.0081 (8)	−0.0018 (8)
C2	0.0185 (9)	0.0135 (9)	0.0185 (9)	−0.0042 (7)	0.0081 (7)	−0.0013 (7)
C3	0.0176 (9)	0.0141 (9)	0.0225 (10)	−0.0018 (7)	0.0089 (8)	0.0008 (7)
C4	0.0164 (9)	0.0132 (9)	0.0329 (12)	−0.0026 (7)	0.0076 (9)	0.0010 (8)
C5	0.0167 (9)	0.0138 (9)	0.0199 (9)	−0.0039 (7)	0.0079 (7)	0.0000 (7)
C6	0.0152 (9)	0.0135 (9)	0.0223 (10)	−0.0030 (7)	0.0065 (7)	−0.0005 (8)
C7	0.0164 (9)	0.0129 (8)	0.0188 (9)	−0.0017 (7)	0.0078 (7)	0.0004 (7)
C8	0.0175 (9)	0.0125 (8)	0.0194 (9)	−0.0023 (7)	0.0082 (7)	−0.0018 (7)
C9	0.0344 (13)	0.0166 (10)	0.0190 (10)	−0.0030 (9)	0.0086 (9)	−0.0002 (8)
C10	0.0388 (14)	0.0178 (10)	0.0235 (11)	−0.0029 (10)	0.0109 (10)	0.0026 (9)
C11	0.0259 (11)	0.0141 (9)	0.0269 (11)	−0.0050 (8)	0.0101 (9)	0.0000 (8)
C12	0.0257 (11)	0.0183 (10)	0.0250 (11)	−0.0085 (9)	0.0067 (9)	−0.0039 (8)
C13	0.0234 (10)	0.0159 (9)	0.0188 (10)	−0.0053 (8)	0.0046 (8)	0.0000 (8)
C14	0.0186 (9)	0.0142 (9)	0.0274 (11)	−0.0018 (8)	0.0078 (8)	0.0001 (8)
C15	0.0270 (4)	0.0249 (4)	0.0211 (4)	0.0066 (3)	0.0066 (3)	0.0059 (3)
C16	0.0270 (4)	0.0249 (4)	0.0211 (4)	0.0066 (3)	0.0066 (3)	0.0059 (3)
C17	0.0270 (4)	0.0249 (4)	0.0211 (4)	0.0066 (3)	0.0066 (3)	0.0059 (3)
C15'	0.0214 (9)	0.0245 (10)	0.0325 (12)	−0.0058 (8)	0.0073 (7)	−0.0081 (8)
C16'	0.0214 (9)	0.0245 (10)	0.0325 (12)	−0.0058 (8)	0.0073 (7)	−0.0081 (8)
C17'	0.0214 (9)	0.0245 (10)	0.0325 (12)	−0.0058 (8)	0.0073 (7)	−0.0081 (8)
C18	0.0178 (9)	0.0148 (9)	0.0215 (10)	−0.0044 (8)	0.0066 (8)	0.0010 (8)
C19	0.0215 (11)	0.0147 (10)	0.0372 (14)	−0.0047 (8)	0.0054 (10)	0.0034 (9)
C20	0.0296 (13)	0.0156 (10)	0.0440 (16)	−0.0043 (10)	0.0038 (11)	0.0061 (10)
C21	0.0369 (14)	0.0214 (12)	0.0292 (13)	−0.0146 (11)	0.0041 (11)	0.0044 (10)
C22	0.0267 (12)	0.0266 (12)	0.0391 (15)	−0.0135 (10)	0.0135 (11)	0.0022 (11)
C23	0.0222 (11)	0.0198 (11)	0.0358 (13)	−0.0079 (9)	0.0126 (10)	−0.0013 (10)
S2	0.0239 (4)	0.0226 (4)	0.0338 (5)	0.0018 (3)	0.0109 (3)	0.0058 (3)
S2'	0.0325 (11)	0.0291 (11)	0.0197 (10)	−0.0075 (8)	0.0035 (7)	−0.0159 (7)
O3	0.0177 (8)	0.0237 (9)	0.0335 (10)	0.0064 (7)	0.0074 (7)	−0.0025 (7)
O4	0.0310 (9)	0.0171 (7)	0.0198 (8)	0.0051 (7)	0.0129 (7)	0.0038 (6)
N2	0.0216 (9)	0.0114 (7)	0.0178 (8)	0.0034 (6)	0.0086 (7)	0.0019 (6)
C24	0.0183 (9)	0.0168 (9)	0.0196 (10)	0.0057 (8)	0.0074 (8)	0.0000 (7)
C25	0.0189 (9)	0.0126 (8)	0.0194 (9)	0.0050 (7)	0.0086 (7)	0.0025 (7)
C26	0.0180 (9)	0.0141 (9)	0.0233 (10)	0.0019 (7)	0.0076 (8)	0.0012 (8)
C27	0.0161 (9)	0.0154 (9)	0.0316 (12)	0.0032 (8)	0.0098 (8)	0.0007 (8)
C28	0.0178 (9)	0.0138 (9)	0.0195 (9)	0.0032 (7)	0.0076 (8)	0.0011 (7)
C29	0.0164 (9)	0.0152 (9)	0.0223 (10)	0.0039 (7)	0.0072 (8)	0.0005 (8)
C30	0.0169 (9)	0.0137 (9)	0.0196 (9)	0.0039 (7)	0.0067 (7)	0.0013 (7)
C31	0.0160 (9)	0.0117 (8)	0.0195 (9)	0.0003 (7)	0.0073 (7)	0.0011 (7)
C32	0.0201 (10)	0.0160 (9)	0.0191 (9)	0.0013 (8)	0.0038 (8)	0.0001 (8)
C33	0.0210 (10)	0.0160 (10)	0.0279 (12)	0.0045 (8)	0.0054 (9)	0.0035 (8)
C34	0.0273 (11)	0.0148 (9)	0.0327 (13)	0.0024 (8)	0.0144 (10)	−0.0040 (9)
C35	0.0425 (15)	0.0198 (11)	0.0200 (11)	0.0008 (10)	0.0128 (10)	−0.0032 (9)
C36	0.0336 (13)	0.0169 (10)	0.0177 (10)	0.0029 (9)	0.0083 (9)	0.0017 (8)
C37	0.0196 (10)	0.0173 (10)	0.0349 (13)	0.0037 (8)	0.0096 (9)	0.0011 (9)
C38	0.0239 (4)	0.0226 (4)	0.0338 (5)	0.0018 (3)	0.0109 (3)	0.0058 (3)
C39	0.0239 (4)	0.0226 (4)	0.0338 (5)	0.0018 (3)	0.0109 (3)	0.0058 (3)
C40	0.0239 (4)	0.0226 (4)	0.0338 (5)	0.0018 (3)	0.0109 (3)	0.0058 (3)
C38'	0.0325 (11)	0.0291 (11)	0.0197 (10)	−0.0075 (8)	0.0035 (7)	−0.0159 (7)
C39'	0.0325 (11)	0.0291 (11)	0.0197 (10)	−0.0075 (8)	0.0035 (7)	−0.0159 (7)
C40'	0.0325 (11)	0.0291 (11)	0.0197 (10)	−0.0075 (8)	0.0035 (7)	−0.0159 (7)
C41	0.0174 (9)	0.0158 (9)	0.0198 (9)	0.0052 (7)	0.0062 (8)	−0.0002 (7)
C42	0.0186 (10)	0.0152 (9)	0.0323 (12)	0.0043 (8)	0.0118 (9)	0.0023 (9)
C43	0.0215 (10)	0.0193 (10)	0.0309 (12)	0.0082 (9)	0.0102 (9)	0.0004 (9)
C44	0.0267 (11)	0.0175 (10)	0.0255 (12)	0.0079 (9)	0.0041 (9)	−0.0012 (9)
C45	0.0266 (11)	0.0145 (9)	0.0268 (11)	0.0011 (8)	0.0065 (9)	0.0000 (8)
C46	0.0187 (10)	0.0177 (10)	0.0249 (11)	0.0020 (8)	0.0075 (8)	−0.0006 (8)

### 5-Oxo-N-phenyl-3-(thiophen-2-yl)-2,3,4,5-tetrahydro-[1,1'-biphenyl]-4-carboxamide Geometric parameters (Å, º)

**Table:**

S1—C14	1.692 (2)	S2—C37	1.686 (2)
S1—C17	1.716 (3)	S2—C40	1.712 (3)
S1'—C14	1.688 (3)	S2'—C37	1.644 (3)
S1'—C17'	1.712 (4)	S2'—C40'	1.722 (4)
O1—C1	1.223 (3)	O3—C24	1.230 (3)
O2—C7	1.227 (3)	O4—C30	1.229 (3)
N1—C7	1.349 (3)	N2—C30	1.352 (3)
N1—C8	1.418 (3)	N2—C31	1.422 (3)
N1—H1N	0.85 (4)	N2—H2N	0.94 (4)
C1—C6	1.455 (3)	C24—C29	1.457 (3)
C1—C2	1.526 (3)	C24—C25	1.516 (3)
C2—C7	1.530 (3)	C25—C30	1.528 (3)
C2—C3	1.543 (3)	C25—C26	1.539 (3)
C2—H2	1.0000	C25—H25	1.0000
C3—C14	1.486 (3)	C26—C37	1.479 (3)
C3—C4	1.535 (3)	C26—C27	1.538 (3)
C3—H3	1.0000	C26—H26	1.0000
C4—C5	1.510 (3)	C27—C28	1.507 (3)
C4—H4A	0.9900	C27—H27A	0.9900
C4—H4B	0.9900	C27—H27B	0.9900
C5—C6	1.354 (3)	C28—C29	1.354 (3)
C5—C18	1.478 (3)	C28—C41	1.472 (3)
C6—H6	0.9500	C29—H29	0.9500
C8—C9	1.395 (3)	C31—C32	1.391 (3)
C8—C13	1.398 (3)	C31—C36	1.392 (3)
C9—C10	1.388 (4)	C32—C33	1.384 (3)
C9—H9	0.9500	C32—H32	0.9500
C10—C11	1.380 (4)	C33—C34	1.385 (4)
C10—H10	0.9500	C33—H33	0.9500
C11—C12	1.397 (4)	C34—C35	1.391 (4)
C11—H11	0.9500	C34—H34	0.9500
C12—C13	1.394 (3)	C35—C36	1.397 (4)
C12—H12	0.9500	C35—H35	0.9500
C13—H13	0.9500	C36—H36	0.9500
C14—C15	1.369 (4)	C37—C38'	1.377 (4)
C14—C15'	1.381 (4)	C37—C38	1.392 (4)
C15—C16	1.440 (4)	C38—C39	1.435 (4)
C15—H15	0.9500	C38—H38	0.9500
C16—C17	1.362 (3)	C39—C40	1.358 (3)
C16—H16	0.9500	C39—H39	0.9500
C17—H17	0.9500	C40—H40	0.9500
C15'—C16'	1.432 (4)	C38'—C39'	1.430 (4)
C15'—H15A	0.9500	C38'—H38A	0.9500
C16'—C17'	1.361 (4)	C39'—C40'	1.360 (4)
C16'—H16A	0.9500	C39'—H39A	0.9500
C17'—H17A	0.9500	C40'—H40A	0.9500
C18—C23	1.393 (3)	C41—C42	1.404 (3)
C18—C19	1.401 (4)	C41—C46	1.407 (3)
C19—C20	1.389 (4)	C42—C43	1.395 (3)
C19—H19	0.9500	C42—H42	0.9500
C20—C21	1.389 (4)	C43—C44	1.386 (4)
C20—H20	0.9500	C43—H43	0.9500
C21—C22	1.381 (5)	C44—C45	1.394 (4)
C21—H21	0.9500	C44—H44	0.9500
C22—C23	1.394 (4)	C45—C46	1.386 (3)
C22—H22	0.9500	C45—H45	0.9500
C23—H23	0.9500	C46—H46	0.9500
			
S1···H2	2.95	C23···H4B	2.57
S2···H25	2.96	C27···H42	2.54
S2···H27B	2.93	C27···H38A	2.97
O2···C13	2.920 (3)	C29···H46	2.65
O4···C36	2.900 (3)	C30···H36	2.79
H16···O1^i^	2.71	C30···H43^i^	2.88
H17A···O1^i^	2.72	C40···H44^iv^	2.84
H22···O1^ii^	2.35	C42···H27A	2.61
O2···H3	2.68	C46···H29	2.59
O2···H13	2.37	H1N···H9	2.32
O2···H2N	1.95 (4)	H1N···H2	2.10
O3···H43^i^	2.42	H2N···H25	2.03
O4···H26	2.67	H4B···H23	1.96
O4···H36	2.38	H6···H19	2.06
H1N···O4^iii^	2.07 (4)	H27A···H42	2.11
C4···H23	2.61	H27B···H38A	2.37
C6···H19	2.62	H29···H46	2.05
C7···H13	2.84	H40···H44^iv^	2.36
C19···H6	2.58		
			
C14—S1—C17	91.85 (14)	C37—S2—C40	94.03 (16)
C14—S1'—C17'	93.6 (3)	C37—S2'—C40'	91.6 (3)
C7—N1—C8	127.3 (2)	C30—N2—C31	126.5 (2)
C7—N1—H1N	115 (3)	C30—N2—H2N	114 (2)
C8—N1—H1N	118 (3)	C31—N2—H2N	119 (2)
O1—C1—C6	121.0 (2)	O3—C24—C29	121.1 (2)
O1—C1—C2	121.7 (2)	O3—C24—C25	120.6 (2)
C6—C1—C2	117.26 (19)	C29—C24—C25	118.20 (19)
C1—C2—C7	110.71 (19)	C24—C25—C30	110.62 (18)
C1—C2—C3	110.18 (18)	C24—C25—C26	112.44 (18)
C7—C2—C3	110.10 (18)	C30—C25—C26	108.80 (19)
C1—C2—H2	108.6	C24—C25—H25	108.3
C7—C2—H2	108.6	C30—C25—H25	108.3
C3—C2—H2	108.6	C26—C25—H25	108.3
C14—C3—C4	112.79 (15)	C37—C26—C27	110.94 (16)
C14—C3—C2	112.29 (16)	C37—C26—C25	111.09 (15)
C4—C3—C2	108.93 (19)	C27—C26—C25	110.6 (2)
C14—C3—H3	107.5	C37—C26—H26	108.0
C4—C3—H3	107.5	C27—C26—H26	108.0
C2—C3—H3	107.5	C25—C26—H26	108.0
C5—C4—C3	113.19 (19)	C28—C27—C26	113.43 (19)
C5—C4—H4A	108.9	C28—C27—H27A	108.9
C3—C4—H4A	108.9	C26—C27—H27A	108.9
C5—C4—H4B	108.9	C28—C27—H27B	108.9
C3—C4—H4B	108.9	C26—C27—H27B	108.9
H4A—C4—H4B	107.8	H27A—C27—H27B	107.7
C6—C5—C18	121.0 (2)	C29—C28—C41	121.8 (2)
C6—C5—C4	119.9 (2)	C29—C28—C27	119.9 (2)
C18—C5—C4	119.08 (19)	C41—C28—C27	118.21 (19)
C5—C6—C1	123.6 (2)	C28—C29—C24	123.8 (2)
C5—C6—H6	118.2	C28—C29—H29	118.1
C1—C6—H6	118.2	C24—C29—H29	118.1
O2—C7—N1	124.8 (2)	O4—C30—N2	124.5 (2)
O2—C7—C2	120.8 (2)	O4—C30—C25	120.8 (2)
N1—C7—C2	114.36 (19)	N2—C30—C25	114.6 (2)
C9—C8—C13	119.7 (2)	C32—C31—C36	120.0 (2)
C9—C8—N1	115.7 (2)	C32—C31—N2	117.2 (2)
C13—C8—N1	124.6 (2)	C36—C31—N2	122.7 (2)
C10—C9—C8	120.4 (2)	C33—C32—C31	120.1 (2)
C10—C9—H9	119.8	C33—C32—H32	119.9
C8—C9—H9	119.8	C31—C32—H32	119.9
C11—C10—C9	120.3 (2)	C32—C33—C34	120.7 (2)
C11—C10—H10	119.8	C32—C33—H33	119.7
C9—C10—H10	119.8	C34—C33—H33	119.7
C10—C11—C12	119.6 (2)	C33—C34—C35	119.1 (2)
C10—C11—H11	120.2	C33—C34—H34	120.4
C12—C11—H11	120.2	C35—C34—H34	120.4
C13—C12—C11	120.7 (2)	C34—C35—C36	120.9 (2)
C13—C12—H12	119.6	C34—C35—H35	119.6
C11—C12—H12	119.6	C36—C35—H35	119.6
C12—C13—C8	119.3 (2)	C31—C36—C35	119.1 (2)
C12—C13—H13	120.4	C31—C36—H36	120.4
C8—C13—H13	120.4	C35—C36—H36	120.4
C15—C14—C3	125.1 (3)	C38'—C37—C26	124.9 (4)
C15'—C14—C3	131.5 (4)	C38—C37—C26	126.3 (3)
C15'—C14—S1'	103.1 (4)	C38'—C37—S2'	109.8 (4)
C3—C14—S1'	125.3 (2)	C26—C37—S2'	125.3 (2)
C15—C14—S1	112.0 (3)	C38—C37—S2	107.7 (3)
C3—C14—S1	122.98 (16)	C26—C37—S2	126.02 (18)
C14—C15—C16	112.8 (4)	C37—C38—C39	116.8 (4)
C14—C15—H15	123.6	C37—C38—H38	121.6
C16—C15—H15	123.6	C39—C38—H38	121.6
C17—C16—C15	110.5 (4)	C40—C39—C38	108.2 (4)
C17—C16—H16	124.7	C40—C39—H39	125.9
C15—C16—H16	124.7	C38—C39—H39	125.9
C16—C17—S1	112.8 (3)	C39—C40—S2	113.3 (3)
C16—C17—H17	123.6	C39—C40—H40	123.3
S1—C17—H17	123.6	S2—C40—H40	123.3
C14—C15'—C16'	125.5 (8)	C37—C38'—C39'	118.2 (7)
C14—C15'—H15A	117.3	C37—C38'—H38A	120.9
C16'—C15'—H15A	117.3	C39'—C38'—H38A	120.9
C17'—C16'—C15'	99.9 (8)	C40'—C39'—C38'	104.0 (8)
C17'—C16'—H16A	130.0	C40'—C39'—H39A	128.0
C15'—C16'—H16A	130.0	C38'—C39'—H39A	128.0
C16'—C17'—S1'	117.9 (7)	C39'—C40'—S2'	116.4 (6)
C16'—C17'—H17A	121.1	C39'—C40'—H40A	121.8
S1'—C17'—H17A	121.1	S2'—C40'—H40A	121.8
C23—C18—C19	118.1 (2)	C42—C41—C46	118.0 (2)
C23—C18—C5	121.0 (2)	C42—C41—C28	120.3 (2)
C19—C18—C5	120.9 (2)	C46—C41—C28	121.7 (2)
C20—C19—C18	121.1 (3)	C43—C42—C41	120.7 (2)
C20—C19—H19	119.5	C43—C42—H42	119.6
C18—C19—H19	119.5	C41—C42—H42	119.6
C19—C20—C21	119.8 (3)	C44—C43—C42	120.5 (2)
C19—C20—H20	120.1	C44—C43—H43	119.8
C21—C20—H20	120.1	C42—C43—H43	119.8
C22—C21—C20	119.9 (2)	C43—C44—C45	119.5 (2)
C22—C21—H21	120.1	C43—C44—H44	120.3
C20—C21—H21	120.1	C45—C44—H44	120.3
C21—C22—C23	120.2 (3)	C46—C45—C44	120.4 (2)
C21—C22—H22	119.9	C46—C45—H45	119.8
C23—C22—H22	119.9	C44—C45—H45	119.8
C18—C23—C22	120.9 (3)	C45—C46—C41	120.9 (2)
C18—C23—H23	119.6	C45—C46—H46	119.6
C22—C23—H23	119.6	C41—C46—H46	119.6
			
O1—C1—C2—C7	25.9 (3)	O3—C24—C25—C30	−35.2 (3)
C6—C1—C2—C7	−156.0 (2)	C29—C24—C25—C30	147.8 (2)
O1—C1—C2—C3	147.9 (2)	O3—C24—C25—C26	−157.1 (2)
C6—C1—C2—C3	−34.0 (3)	C29—C24—C25—C26	26.0 (3)
C1—C2—C3—C14	−177.06 (17)	C24—C25—C26—C37	−173.58 (18)
C7—C2—C3—C14	−54.7 (2)	C30—C25—C26—C37	63.5 (2)
C1—C2—C3—C4	57.3 (2)	C24—C25—C26—C27	−49.9 (3)
C7—C2—C3—C4	179.64 (19)	C30—C25—C26—C27	−172.79 (19)
C14—C3—C4—C5	−177.59 (19)	C37—C26—C27—C28	173.80 (19)
C2—C3—C4—C5	−52.2 (3)	C25—C26—C27—C28	50.0 (3)
C3—C4—C5—C6	22.4 (3)	C26—C27—C28—C29	−25.5 (3)
C3—C4—C5—C18	−157.9 (2)	C26—C27—C28—C41	156.6 (2)
C18—C5—C6—C1	−176.4 (2)	C41—C28—C29—C24	177.4 (2)
C4—C5—C6—C1	3.3 (4)	C27—C28—C29—C24	−0.4 (4)
O1—C1—C6—C5	−178.8 (2)	O3—C24—C29—C28	−176.9 (2)
C2—C1—C6—C5	3.1 (3)	C25—C24—C29—C28	0.1 (3)
C8—N1—C7—O2	6.8 (4)	C31—N2—C30—O4	−3.7 (4)
C8—N1—C7—C2	−171.5 (2)	C31—N2—C30—C25	174.1 (2)
C1—C2—C7—O2	71.4 (3)	C24—C25—C30—O4	−60.9 (3)
C3—C2—C7—O2	−50.7 (3)	C26—C25—C30—O4	63.1 (3)
C1—C2—C7—N1	−110.2 (2)	C24—C25—C30—N2	121.2 (2)
C3—C2—C7—N1	127.7 (2)	C26—C25—C30—N2	−114.8 (2)
C7—N1—C8—C9	158.7 (2)	C30—N2—C31—C32	157.3 (2)
C7—N1—C8—C13	−22.4 (4)	C30—N2—C31—C36	−25.8 (4)
C13—C8—C9—C10	0.3 (4)	C36—C31—C32—C33	1.8 (4)
N1—C8—C9—C10	179.3 (2)	N2—C31—C32—C33	178.9 (2)
C8—C9—C10—C11	−0.3 (4)	C31—C32—C33—C34	−0.9 (4)
C9—C10—C11—C12	0.2 (4)	C32—C33—C34—C35	−0.8 (4)
C10—C11—C12—C13	−0.2 (4)	C33—C34—C35—C36	1.5 (4)
C11—C12—C13—C8	0.2 (4)	C32—C31—C36—C35	−1.1 (4)
C9—C8—C13—C12	−0.2 (4)	N2—C31—C36—C35	−178.0 (2)
N1—C8—C13—C12	−179.2 (2)	C34—C35—C36—C31	−0.6 (4)
C4—C3—C14—C15	−111.8 (2)	C27—C26—C37—C38'	−57.0 (2)
C2—C3—C14—C15	124.67 (19)	C25—C26—C37—C38'	66.5 (2)
C4—C3—C14—C15'	69.4 (2)	C27—C26—C37—C38	119.5 (2)
C2—C3—C14—C15'	−54.2 (2)	C25—C26—C37—C38	−117.0 (2)
C4—C3—C14—S1'	−110.7 (2)	C27—C26—C37—S2'	123.1 (2)
C2—C3—C14—S1'	125.8 (2)	C25—C26—C37—S2'	−113.4 (2)
C4—C3—C14—S1	68.23 (18)	C27—C26—C37—S2	−60.68 (18)
C2—C3—C14—S1	−55.32 (17)	C25—C26—C37—S2	62.81 (17)
C17'—S1'—C14—C15'	−0.03 (12)	C40'—S2'—C37—C38'	0.00 (12)
C17'—S1'—C14—C3	−179.97 (7)	C40'—S2'—C37—C26	179.91 (7)
C17—S1—C14—C15	−0.02 (11)	C40—S2—C37—C38	−0.36 (11)
C17—S1—C14—C3	179.97 (6)	C40—S2—C37—C26	179.79 (7)
C3—C14—C15—C16	179.94 (10)	C26—C37—C38—C39	−179.62 (11)
S1—C14—C15—C16	−0.07 (18)	S2—C37—C38—C39	0.54 (18)
C14—C15—C16—C17	0.1 (2)	C37—C38—C39—C40	−0.5 (2)
C15—C16—C17—S1	−0.2 (2)	C38—C39—C40—S2	0.2 (2)
C14—S1—C17—C16	0.11 (14)	C37—S2—C40—C39	0.12 (15)
C3—C14—C15'—C16'	−179.92 (14)	C26—C37—C38'—C39'	−179.91 (13)
S1'—C14—C15'—C16'	0.2 (2)	S2'—C37—C38'—C39'	0.0 (2)
C14—C15'—C16'—C17'	−0.2 (3)	C37—C38'—C39'—C40'	0.0 (3)
C15'—C16'—C17'—S1'	0.2 (2)	C38'—C39'—C40'—S2'	0.0 (2)
C14—S1'—C17'—C16'	−0.08 (17)	C37—S2'—C40'—C39'	0.00 (17)
C6—C5—C18—C23	−161.9 (2)	C29—C28—C41—C42	−166.2 (2)
C4—C5—C18—C23	18.4 (3)	C27—C28—C41—C42	11.7 (3)
C6—C5—C18—C19	18.0 (4)	C29—C28—C41—C46	14.1 (4)
C4—C5—C18—C19	−161.7 (2)	C27—C28—C41—C46	−168.0 (2)
C23—C18—C19—C20	1.3 (4)	C46—C41—C42—C43	−1.0 (4)
C5—C18—C19—C20	−178.6 (3)	C28—C41—C42—C43	179.3 (2)
C18—C19—C20—C21	−0.3 (5)	C41—C42—C43—C44	1.1 (4)
C19—C20—C21—C22	−0.6 (5)	C42—C43—C44—C45	−0.6 (4)
C20—C21—C22—C23	0.5 (5)	C43—C44—C45—C46	−0.1 (4)
C19—C18—C23—C22	−1.4 (4)	C44—C45—C46—C41	0.2 (4)
C5—C18—C23—C22	178.5 (3)	C42—C41—C46—C45	0.3 (4)
C21—C22—C23—C18	0.5 (5)	C28—C41—C46—C45	−180.0 (2)

Symmetry codes: (i) *x*+1/2, *y*−1/2, *z*; (ii) *x*+1/2, *y*+1/2, *z*; (iii) *x*, −*y*+1, *z*+1/2; (iv) *x*, *y*−1, *z*.

### 5-Oxo-N-phenyl-3-(thiophen-2-yl)-2,3,4,5-tetrahydro-[1,1'-biphenyl]-4-carboxamide Hydrogen-bond geometry (Å, º)

Cg1, Cg2, Cg7 and Cg10 are the centroids of the S1/C14···C17, S2/C37···C49, C18···C23 and C41···C46 rings, respectively.

**Table:**

*D*—H···*A*	*D*—H	H···*A*	*D*···*A*	*D*—H···*A*
N1—H1*N*···O4^iii^	0.85 (4)	2.07 (4)	2.921 (3)	173 (4)
N2—H2*N*···O2	0.94 (4)	1.95 (4)	2.871 (3)	167 (3)
C11—H11···*Cg*1^v^	0.95	2.71	3.468 (3)	137
C12—H12···*Cg*10^vi^	0.95	2.87	3.772 (3)	159
C34—H34···*Cg*2^i^	0.95	2.78	3.546 (3)	139
C38—H38···*Cg*7^v^	0.95	2.81	3.603 (5)	142

Symmetry codes: (i) *x*+1/2, *y*−1/2, *z*; (iii) *x*, −*y*+1, *z*+1/2; (v) *x*−1/2, *y*+1/2, *z*; (vi) *x*, −*y*+2, *z*−1/2.
